# PRKD3 Overexpression May Improve Survival and Suppresses Proliferation in Colorectal Cancer

**DOI:** 10.1002/cnr2.70543

**Published:** 2026-04-14

**Authors:** Bin Zhang, Chen Chang, Jingli Chen, Yue Cao, Yu Chang, Lele Liu, Wensheng Li, Guorong Wang

**Affiliations:** ^1^ First Department of General Surgery Shaanxi Provincial People's Hospital Xi'an China; ^2^ Xi'an Medical University Xi'an China; ^3^ The Third Affiliated Hospital of The School of Medicine, Xi'an Jiaotong University Xi'an China; ^4^ Department of Pathology Shaanxi Provincial People's Hospital Xi'an China

**Keywords:** diagnosis, molecular markers, PRKD3, prognosiscolorectal cancer

## Abstract

**Objective:**

The study aimed to explore PRKD3 protein expression in colorectal cancer and its clinical implications.

**Methods:**

PRKD3 expression was assessed in 189 paired colorectal cancer tissues and their corresponding adjacent non‐cancerous counterparts using tissue microarray‐based immunohistochemistry. The associations of PRKD3 expression with clinicopathological parameters and patient prognosis were analyzed. PRKD3 mRNA levels were quantified by quantitative real‐time polymerase chain reaction (qPCR), and its protein expression in colorectal cancer cell lines was detected by Western blot analysis. To investigate the functional role of PRKD3 in cell proliferation, its expression was knocked down using siRNA transfection, followed by proliferation assays.

**Results:**

Immunohistochemical analysis revealed that PRKD3 protein was predominantly localized in the cytoplasm of both colorectal cancer and adjacent normal epithelial cells (sampled from > 5 cm beyond the tumor margin). The positive expression rate was 51.8% (72/139) in adjacent normal tissues and 68.7% (114/166) in colorectal cancer tissues, showing a statistically significant increase in cancer tissues (*p* < 0.05). PRKD3 expression was significantly associated with the clinical diagnosis (colon vs. rectal cancer) (*p* < 0.05). Kaplan–Meier survival analysis demonstrated that patients with high PRKD3 expression had a significantly longer postoperative survival than those with low expression (*p* < 0.05). Furthermore, cell proliferation assays showed that knockdown of PRKD3 significantly enhanced proliferation ability compared with the control group.

**Conclusion:**

Taken together, the positive expression of PRKD3 may be associated with better prognosis. These findings suggest that PRKD3 may be involved in the development and progression of colorectal cancer and could represent a potential prognostic biomarker and therapeutic target for this disease.

## 1 Introduction

Colorectal cancer (CRC) is a prevalent gastrointestinal malignancy. According to the National Cancer Institute (NCI), CRC is the third most commonly diagnosed cancer and the second leading cause of cancer‐related death globally [1]. According to the latest China Cancer Statistics Report (2022), CRC ranks as the fourth most common malignancy and the fourth leading cause of cancer‐related death in China, with its incidence showing an increasing trend [2]. Therefore, identifying more effective biomarkers to elucidate the pathogenesis of CRC remains a primary challenge and research focus, given its significant threat to public health both in China and globally.

The PRKD3 gene, which encodes the protein kinase D3, is positioned on the human chromosome 2p22.2 and encompasses 22 exons. As a member of the protein kinase D family, it functions as a serine/threonine kinase. This gene was initially identified and characterized by Akiko Hayashi et al. in 1999 [3]. The PRKD protein family has been implicated in various biological processes, including cell growth, invasion, angiogenesis, protein transport, transcriptional regulation, and epithelial to mesenchymal transition [4]. However, the role of PRKD3 in CRC remains poorly understood.

We employed tissue microarray and immunohistochemistry to assess PRKD3 protein expression in CRC and adjacent normal tissues. We further investigated the association between PRKD3 expression levels and the clinicopathological characteristics and prognosis of CRC patients.

## 2 Materials and Methods

### 2.1 Databases Analysis

A dataset comprising 100 CRC cases and their clinicopathological indicators was sourced from The Cancer Genome Atlas (TCGA). PRKD3 mRNA expression was compared between normal and tumor tissues. Correlation analysis was performed to evaluate the relationship of PRKD3 expression with a range of clinicopathological parameters—including lymphatic invasion, history of colon polyps, overall survival (OS) events, pathologic T/N/M stages, tumor type, and pathologic stage—as well as patient prognosis.

### 2.2 Sample Collection

This study was approved by the Ethics Committee of Shaanxi Provincial People's Hospital (No. 2023‐R057). We retrospectively reviewed archival paraffin‐embedded tissues from 189 CRC cases, along with their morphologically normal adjacent tissues (located > 5 cm from the tumor margin). All tissues were residual diagnostic specimens from patients who underwent resection at our hospital between January 2017 and December 2018. All cases were confirmed by histopathological examination and staged according to the AJCC 7th edition CRC staging system. Patients were followed up for 5 years [5].

This study initially enrolled 189 CRC cases with complete clinicopathological data and systematic follow‐up. Postoperative paraffin‐embedded specimens were collected from all patients. However, some tissue sections were excluded from subsequent analysis for the following reasons: absence of representative tumor regions or normal resection margins in the sampled specimens and tissue detachment during immunohistochemical staining, which precluded reliable scoring. Consequently, the final analyzable cohort consisted of 166 CRC tissues and 139 paired adjacent normal tissues, all with complete datasets for clinicopathological parameters and follow‐up.

### 2.3 Sample Inclusion and Exclusion Criteria

Inclusion Criteria: (1) Patients with primary colorectal cancer confirmed by histopathology who underwent radical resection. (2) No history of preoperative radiotherapy or chemotherapy. (3) Availability of complete clinicopathological and follow‐up records.

Exclusion Criteria: (1) History of any other malignant tumor. (2) Administration of preoperative radiotherapy or chemotherapy. (3) Pathologically confirmed metastatic colorectal cancer. (4) Death from causes unrelated to colorectal cancer. (5) Withdrawal of or failure to provide informed consent.

### 2.4 Main Reagents

Immunohistochemical staining was performed using a primary antibody against PRKD3 (Proteintech; Cat. # 12785‐1‐AP) at a dilution of 1:500. The PV‐8000 polymer detection system and DAB chromogen (Zhongshan Jinqiao Biotechnology; Cat. # PV‐8000‐1 and ZLI‐9017, respectively) were used for visualization according to the manufacturer's instructions.

### 2.5 Immunohistochemical Detection

A tissue microarray (TMA) of CRC was constructed. For each case, three representative tumor cores and two matched adjacent normal tissue cores were sampled. Subsequently, 4‐μm‐thick sections were cut from the TMA block for immunohistochemical analysis [6]. Immunohistochemical staining for PRKD3 was performed according to standard protocols. Briefly, formalin‐fixed, paraffin‐embedded sections were deparaffinized, rehydrated, and subjected to antigen retrieval in sodium citrate buffer using a microwave oven. Endogenous peroxidase activity was quenched with 3% hydrogen peroxide. The sections were then incubated with the primary antibody against PRKD3 (1:500) overnight at 4°C, followed by incubation with the PV‐8000 polymer detection system. Signal was developed using DAB, and the nuclei were counterstained with hematoxylin. Finally, the slides were dehydrated, cleared, and mounted.

All stained slides were digitally scanned using a high‐resolution whole‐slide scanner. PRKD3 expression was evaluated independently by two pathologists who were blinded to the clinical data. A semi‐quantitative histoscore (H‐score) was calculated based on the staining intensity (0–3) and the percentage of positive tumor cells (0–100%) [7].

Two experienced pathologists independently scored each tissue section without prior knowledge of the clinical information or each other's assessment results. The scoring criteria were as follows: Staining intensity (*I*) was graded on a 4‐point scale: 0 (no staining), 1 (light yellow), 2 (brownish yellow), and 3 (brown). The percentage of positive cells (*P*) was estimated as follows: 0 (< 1%), 1 (1%–20%), 2 (21%–50%), and 3 (51%–100%). The final immunohistochemistry (IHC) score was calculated as *I* × *P*, yielding a theoretical range of 0–9.

For survival or correlation analysis, the continuous IHC scores were dichotomized into categorical variables. Using the optimal cutoff value determined by X‐tile software, patients were stratified into a low‐expression group (IHC score ≤ 4.0) and a high‐expression group (IHC score > 4.0). Prior to evaluation, all tissue sections were randomly re‐coded to conceal patient identifiers, pathological diagnoses, and clinical outcomes. Throughout the scoring process, the evaluators remained blinded to the sample group (cancer vs. normal), patient prognosis, and other clinicopathological parameters, thereby ensuring objective assessment.

In cases where significant discrepancies were observed between the two IHC scores, a third senior pathologist with extensive expertise re‐evaluated the corresponding sections. The results were then reconciled to minimize data error arising from inter‐observer variability.

### 2.6 Quantitative Real‐Time PCR

A separate cohort of 12 paired colorectal cancer and adjacent normal tissue specimens was obtained from Shaanxi Provincial People's Hospital. The patient inclusion and exclusion criteria were identical to those described for the immunohistochemistry study cohort.

Total RNA was extracted from these tissues using the TIANGEN RNA extraction kit (DP419). Subsequently, cDNA was synthesized from 1 μg of total RNA using the AG reverse transcription kit (AG11706). Quantitative real‐time PCR (qRT‐PCR) was performed using the AG SYBR Green qPCR Master Mix (AG11701), strictly following the manufacturers' protocols. The relative expression level of PRKD3 mRNA was normalized to that of β‐actin and calculated using the 2^−ΔΔCt^ method.

The primer sequences used were as follows:

*PRKD3* forward: 5′‐GAGCCTGCCACTGCTAACTA‐3′.

*PRKD3* reverse: 5′‐GTCCTCATTTTCATTCTGGGGG‐3′.

*β‐actin* primers were commercially obtained from AG (AG11722).

### 2.7 Cell Culture

Four human colorectal cancer cell lines—HT29, HCT116, SW620, and DLD1—were purchased from Nanjing Kebai Biotechnology Co. Ltd.

SW620 and DLD1 cells were maintained in high‐glucose Dulbecco's Modified Eagle Medium (DMEM), while HT29 and HCT116 cells were cultured in RPMI‐1640 medium. Both media were supplemented with 10% fetal bovine serum (Newzerum Ltd.) and 1% penicillin/streptomycin (Pricella). All cells were cultured at 37°C in a humidified incubator with 5% CO_2_. The medium was refreshed every 2–3 days.

Cells were passaged or harvested for experiments when they reached approximately 80% confluence (logarithmic growth phase) using 0.25% trypsin‐EDTA (Solarbio).

All cell lines were routinely tested and confirmed to be free of mycoplasma contamination.

### 2.8 Cell Transfection

SW620 and DLD1 cells were seeded in 6‐well plates at an appropriate density (e.g., 2 × 10^5^ cells per well) and allowed to adhere overnight. Transient transfection was carried out using Lipofectamine 2000 Transfection Reagent (Thermo Fisher Scientific) according to the manufacturer's instructions. Briefly, specific siRNAs targeting PRKD3 (si‐PRKD3‐1 and si‐PRKD3‐2) or a negative control siRNA (si‐NC) (all synthesized by Beijing Tianyi Huiyuan Co.) were complexed with Lipofectamine 2000 in Opti‐MEM Reduced Serum Medium (Thermo Fisher Scientific) and then added to the cells.

The siRNA sequences were as follows:

si‐NC: sense 5′‐UUCUCCGAACGUGUCACGUTT‐3′, antisense 5′‐ACGUGACACGUUCGGAGAATT‐3′.

si‐PRKD3‐1: sense 5′‐GGAGAGUGUUACCAUUGAADTDT‐3′, antisense 5′‐UUCAAUGGUAACACUCUCCDTDT‐3′.

si‐PRKD3‐2: sense 5′‐CGAAUAUGUCAGUACUGCAADTDT‐3′, antisense 5′‐UUGCAGUACUGACAUAUCGDTDT‐3′.

After 4–6 h of incubation, the transfection mixture was replaced with complete growth medium. Cells were cultured for an additional 48 h and then harvested for Western blot analysis.

### 2.9 Western Blot

Total cellular proteins were extracted using a homemade RIPA lysis buffer (50 mM Tris‐HCl, pH 7.4, 150 mM NaCl, 1% NP‐40, 0.1% SDS) supplemented with protease inhibitors. Protein concentrations were determined using a BCA Protein Assay Kit (Solarbio).

For Western blot analysis, equal amounts of protein (25 μg per lane) were separated by SDS‐PAGE on gels prepared with a PAGE Gel Fast Preparation Kit (EpiZyme) and then electrophoretically transferred onto nitrocellulose membranes. The membranes were blocked with 5% non‐fat milk in TBST for 1 h at room temperature and subsequently incubated overnight at 4°C with the following primary antibodies: rabbit monoclonal anti‐PRKD3 (1:500; Cell Signaling Technology, #5655S) and mouse monoclonal anti‐β‐actin (1:1000; Proteintech, #66009‐1‐Ig).

After washing, the membranes were incubated for 1 h at room temperature with horseradish peroxidase (HRP)‐conjugated secondary antibodies: goat anti‐rabbit IgG (1:1000; ORIGENE, #ZB‐5301) and goat anti‐mouse IgG (1:1000; CWBIO, #CW0102S). Protein bands were detected using an enhanced chemiluminescence (ECL) substrate and imaged with a FluorChem FC2 system (Alpha Innotech). Band intensities were quantified using ImageJ software, with PRKD3 signals normalized to β‐actin.

### 2.10 Cell Proliferation Assay

The effect of PRKD3 knockdown on cell proliferation was assessed following siRNA transfection. Cells were seeded in 96‐well plates at a density of 2000 per well, and cell viability was measured every 24 h using the Cell Counting Kit‐8 assay (cat:C0038; Beyotime).

### 2.11 Statistical Analysis

Statistical analyses were performed using SPSS software (version 26.0) and GraphPad Prism (version 8.0.2). The association between PRKD3 expression levels and clinicopathological characteristics was assessed using the Chi‐square (*χ* ^2^) test or Fisher's exact test, as appropriate. Survival analysis was conducted using the Kaplan–Meier method, and differences between groups were compared with the log‐rank test. Univariate and multivariate Cox proportional hazards regression models were employed to identify independent prognostic factors among the clinicopathological variables and PRKD3 expression levels.

For comparisons between two groups of continuous data, Student's *t*‐test was used. For comparisons among more than two groups, one‐way analysis of variance (ANOVA) was applied, followed by Dunnett's post hoc test for multiple comparisons. A two‐sided *p* value < 0.05 was considered statistically significant.

## 3 Results

### 3.1 Expression Level of PRKD3 in the Database

**FIGURE 1:**
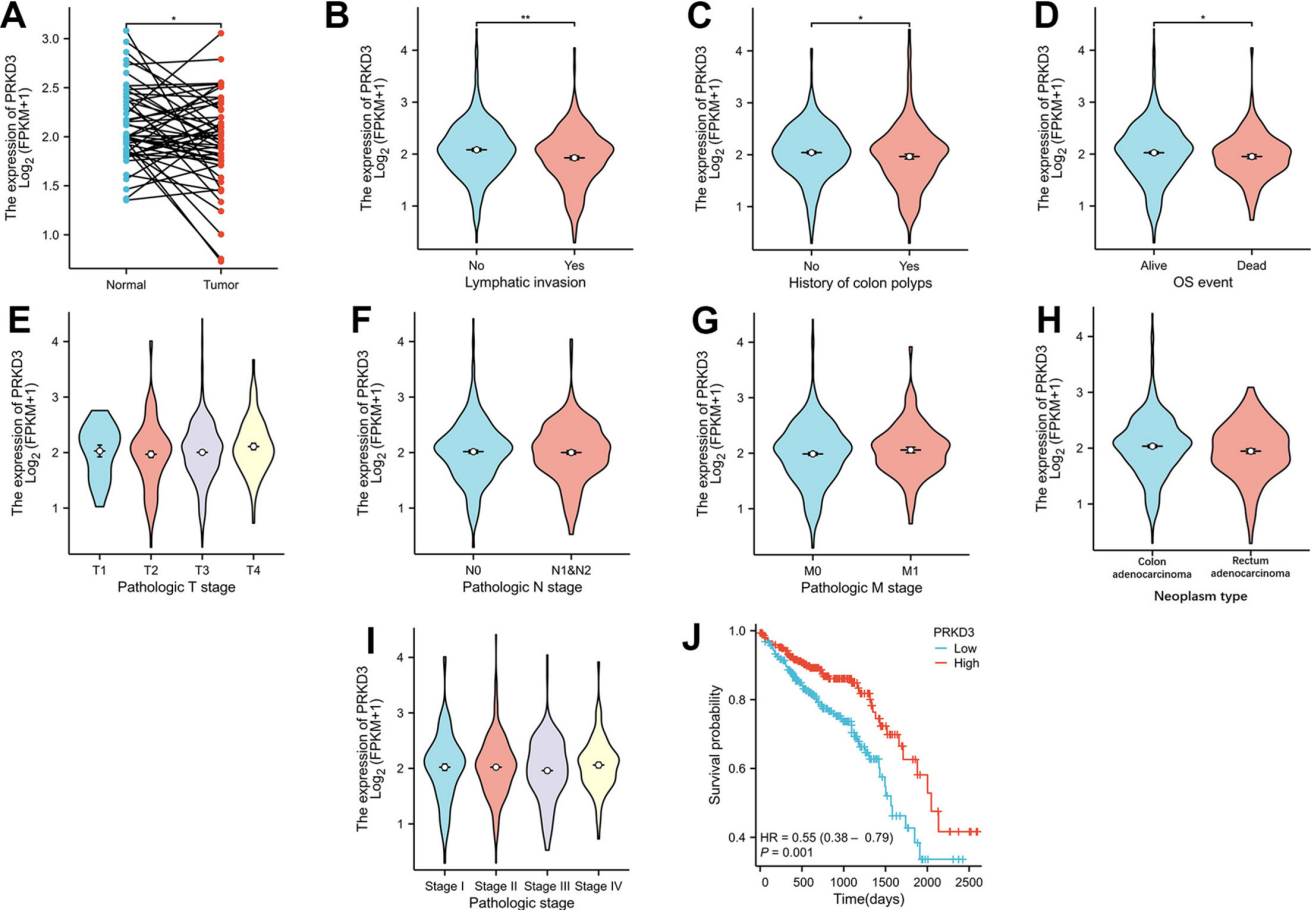
Expression of PRKD3 mRNA in TCGA database. (A) The expression of PRKD3 mRNA in adjacent normal tissues and CRC tissues (*p* < 0.05). (B–I) Correlation analysis between PRKD3 mRNA expression level and clinicopathological parameters in CRC. (B) Lymphatic invasion (*p* < 0.01). (C) History of colonic polyps (*p* < 0.05). (D) OS event (*p* < 0.05). (E) Pathologic T stage. (F) Pathologic N stage. (G) Pathologic M stage. (H) Tumor type (colon adenocarcinoma vs. rectal adenocarcinoma); (I) pathological stage. (J) The relationship between PRKD3 mRNA level and OS of patients with CRC (*p* = 0.018). *p < 0.05; **p < 0.01.

The expression level of PRKD3 mRNA in CRC tissues was found to be significantly lower compared to normal colorectal tissues in the TCGA database analysis (*p* < 0.05, Figure 1A). Further examination of the correlation between PRKD3 mRNA expression and clinicopathological parameters revealed significant associations with lymphatic vessel invasion (*p* < 0.01; Figure 1B), history of colon polyps (*p* < 0.05; Figure 1C), OS events (*p* < 0.05; Figure 1D), and patient survival prognosis (*p* = 0.001; Figure 1J). However, no significant correlations were observed with pathologic T stage, pathologic N stage, pathologic M stage, tumor type (colon cancer vs. rectal cancer), and pathological stage (p > 0.05; Figure 1E–I).

### 3.2 High Expression of PRKD3 in CRC Tissues

PRKD3 protein expression was assessed by IHC in 189 paired CRC tissues and adjacent normal tissues. After excluding samples with missing values due to tissue loss or inadequate staining, PRKD3 immunoreactivity was primarily localized to the cytoplasm.

**FIGURE 2:**
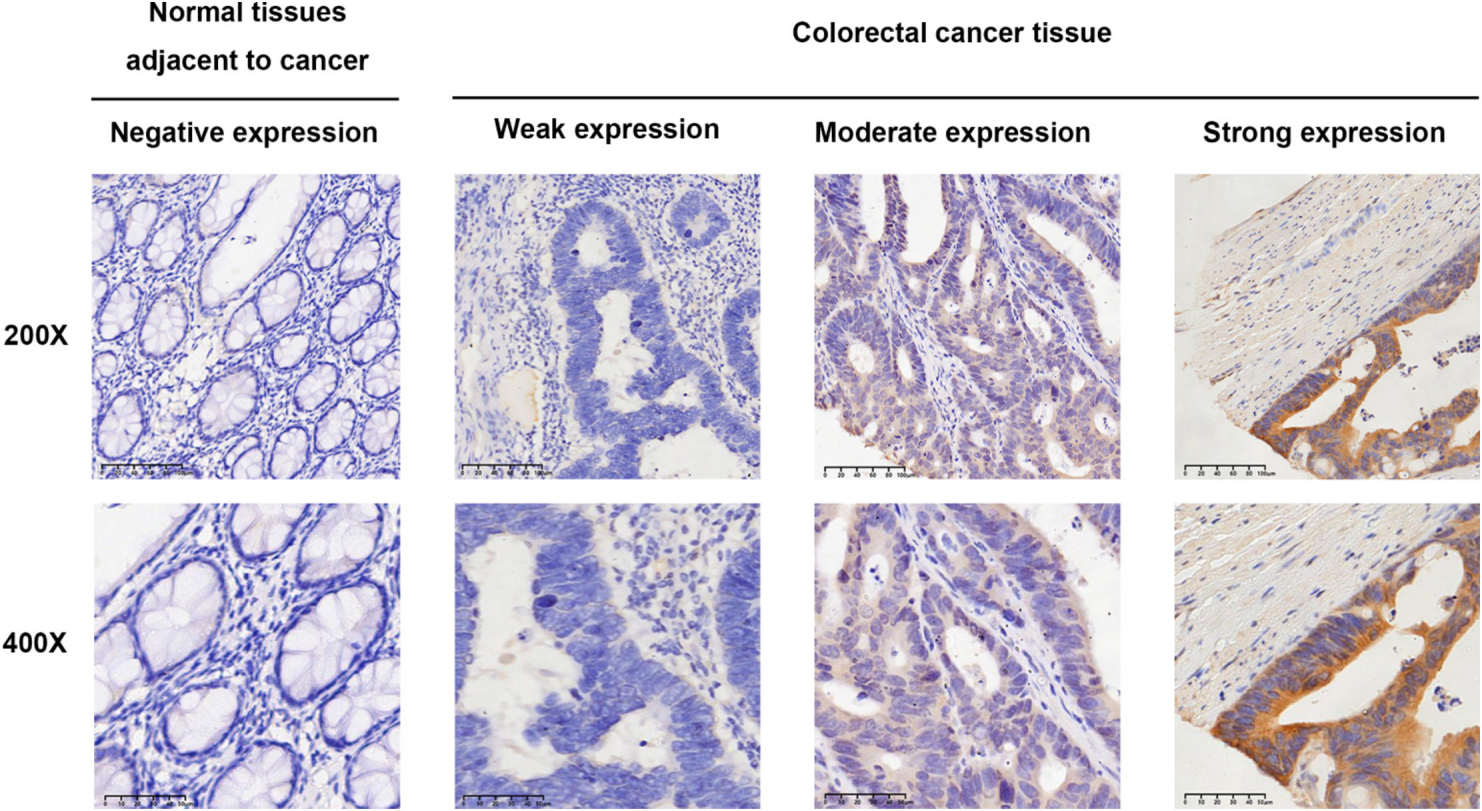
Immunohistochemistry detection of PRKD3 protein expression in CRC tissues.

**FIGURE 3:**
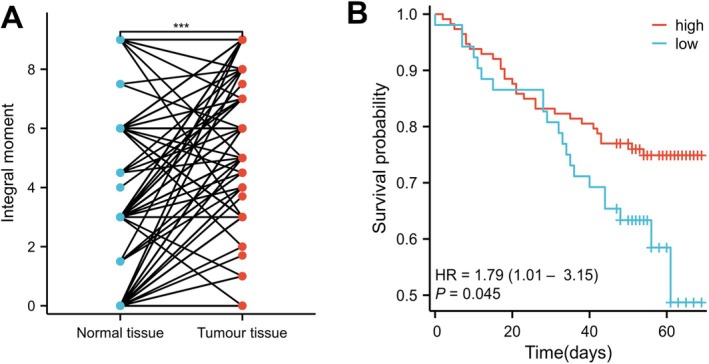
(A) Comparison of PRKD3 protein expression levels between CRC and adjacent normal tissues in 124 cases with complete information. (B) Kaplan–Meier survival curves based on PRKD3 IHC results in CRC. *p < 0.05; **p < 0.01; ***p < 0.001; ****p < 0.0001.

The positive expression rate of PRKD3 was significantly higher in CRC tissues (68.7%, 114/166) than in adjacent normal colorectal epithelium (51.8%, 72/139) (*p* < 0.05; Figures 2, 3A, and Table 1).

**TABLE 1:** Expression of PRKD3 protein in colorectal cancer and adjacent normal epithelial tissues.

Tissue	PRKD3		Total	*p*
	Number of negative cases (%)	Number of positive cases (%)		
Colorectal cancer	52 (31.3%)	114 (68.7%)	166	0.003
Normal tissues adjacent to cancer	67 (48.2%)	72 (51.8%)	139	—

### 3.3 Relationship Between PRKD3 Expression and Clinicopathological Parameters in CRC

The association between PRKD3 protein expression (as determined by IHC) and clinicopathological characteristics was analyzed in the cohort of CRC patients. As summarized in Table 2, PRKD3 expression showed a significant association with the clinical subtype of CRC (*p* = 0.049).

**TABLE 2:** Relationship between PRKD3 protein expression and clinicopathological parameters.

Indicators	*n*	PRKD3		
		Number of negative cases	Number of positive cases	*p*
Sex (*n*)				0.868
Male	91	29	62	
Female	75	23	52	
Age 1 (*n*)				0.721
≥ 50	153	49	104	
< 50	13	3	10	
Age 2 (*n*)				0.858
≥ 60	123	39	84	
< 60	43	13	30	
Clinical diagnosis (*n*)				0.049
Colon cancer	89	22	67	
Rectal cancer	77	30	47	
Clinical diagnosis 2 (*n*)				0.020
Left‐colon cancer	43	7	36	
Right‐colon cancer	41	11	30	
Rectal cancer	77	31	46	
Maximum tumor diameter (*n*)				0.198
< 5 cm	**103**	36	67	
≥ 5 cm	**63**	16	47	
Degree of differentiation (*n*)				0.495
Low	2	0	2	
Medium	123	41	82	
High	40	11	29	
T stage (*n*)				0.934
T1	5	2	3	
T2	19	5	14	
T3	55	17	38	
T4	87	28	59	
N stage (*n*)				0.357
N1–N2	87	30	57	
N0	79	22	57	
M stage (*n*)				0.361
M0	161	49	112	
M1	5	3	2	
Clinical staging (*n*)				0.589
I	15	4	11	
II	69	21	48	
III	79	25	54	
IV	3	2	1	
MMR status (*n*)				0.087
dMMR	60	14	46	
pMMR	102	37	65	
Nerve invasion (*n*)				0.382
No	30	13	17	
Yes	90	31	59	
Vascular invasion (*n*)				0.893
No	40	14	26	
Yes	80	29	51	

When colon cancer cases were further stratified into left‐sided and right‐sided subtypes, PRKD3 expression demonstrated a more pronounced association with the refined diagnostic categories (left‐sided colon cancer, right‐sided colon cancer, and rectal cancer) (*p* = 0.02).

In contrast, PRKD3 expression was not significantly associated with other parameters, including patient sex, age, tumor differentiation grade, TNM stage, clinical stage, maximum tumor diameter, mismatch repair (MMR) status, perineural invasion, or vascular invasion (all *p* > 0.05).

### 3.4 Relationship Between PRKD3 Expression and the Prognosis of Patients With CRC

Univariate Cox proportional hazards regression analysis identified PRKD3 protein expression level, N stage, and clinical stage as factors significantly associated with patient survival time (all *p* < 0.05; Table 3). However, in the subsequent multivariate Cox regression model incorporating these variables, none remained an independent prognostic factor (all *p* > 0.05; Table 3).

**TABLE 3:** Univariate and multivariate COX regression analysis of postoperative survival time in patients with colorectal cancer.

Factors	Univariate analysis		Multivariate analysis	
	*p*	HR (95% CI)	*p*	HR (95% CI%)
Sex (male, female)	0.539	0.839 (0.479–1.469)	—	—
Age 1 (≥ 50, < 50)	0.564	1.41 (0.438–4.536)	—	—
Age 2 (≥ 60, < 60)	0.276	1.471 (0.734–2.948)	—	—
T stage (T1, T2, T3, and T4)	0.186	1.295 (0.883–1.898)	—	—
N stage (N0, N1 + N2)	0.002	2.73 (1.468–5.077)	0.868	0.841 (0.110–6.460)
M stage (M0, M1)	0.47	1.684 (0.409–6.941)	—	—
Clinical staging (I + II; III + IV)	0.001	2.874 (1.564–5.281)	0.24	3.307 (0.449–24.340)
Degree of differentiation (low, moderate and high differentiation)	0.24	1.417 (0.792–2.537)	—	—
Tumor type (colon, rectum)	0.818	1.034 (0.780–1.370)	—	—
Tumor location (left and right colon; rectum)	0.793	0.956 (0.683–1.338)	—	—
MMR status (dMMR; pMMR)	0.584	1.177 (0.657–2.108)	—	—
PRKD3 expression (low, high)	0.042	0.554 (0.314–0.978)	0.056	0.573 (0.324–1.015)

Abbreviations: CI, confidence interval; HR, hazard ratio.

However, the PRKD3 protein expression level (*p* = 0.056; Table 3) approached the threshold, suggesting that PRKD3 expression may be associated with better prognosis in CRC patients. Further investigations with a larger sample size may help reveal possible differences.

### 3.5 Protein and mRNA Expression of PRKD3 in Paired Tissues

To further validate our findings, we performed immunohistochemical (IHC) staining and quantitative real‐time PCR (qPCR) on an independent set of 12 paired CRC and adjacent normal tissues.

**FIGURE 4:**
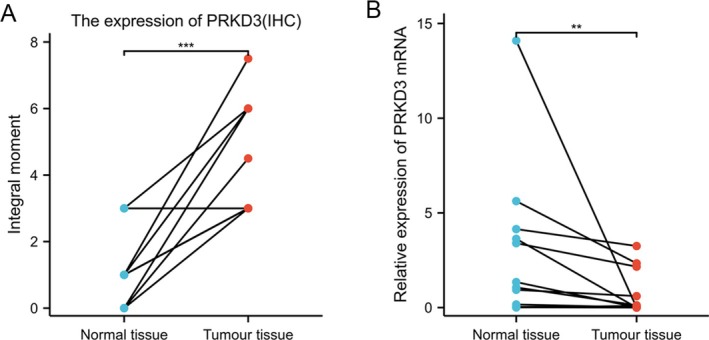
(A) Comparison of PRKD3 protein expression levels in 12 paired colorectal cancer and adjacent normal tissues. (B) Comparison of PRKD3 mRNA expression levels in 12 paired colorectal cancer and adjacent normal tissues. *p < 0.05; **p < 0.01; ***p < 0.001; ****p < 0.0001.

Consistent with the IHC results from our primary cohort (Figures 2 and 3), PRKD3 protein expression was significantly higher in CRC tissues than in matched normal tissues (*p* < 0.05; Figure 4A).

Conversely, and in agreement with the TCGA database analysis (Figure 1A), qPCR analysis revealed that PRKD3 mRNA expression was significantly lower in CRC tissues compared to their normal counterparts (*p* < 0.05; Figure 4B).

### 3.6 Protein Expression of PRKD3 in Colorectal Cancer Cell Lines

**FIGURE 5:**
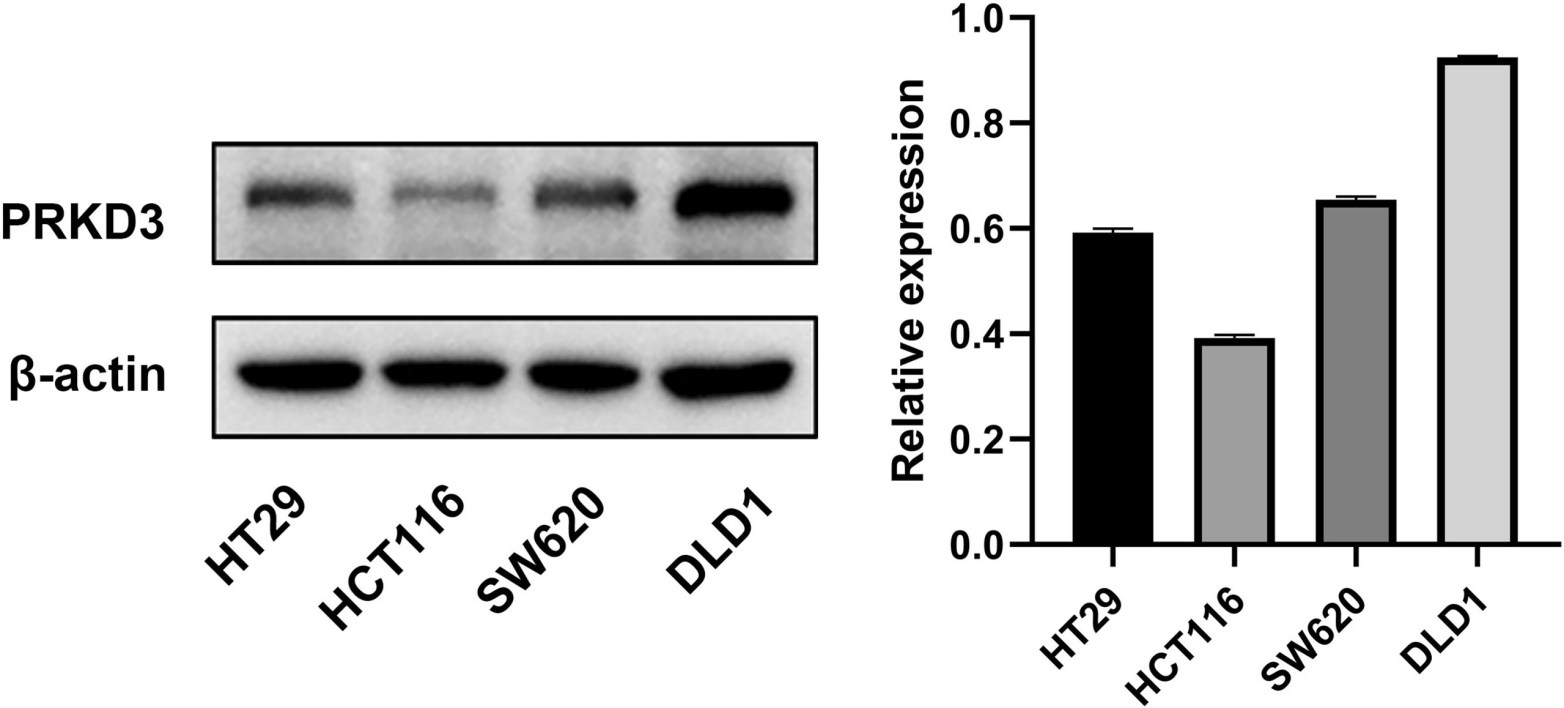
Western blot analysis of PRKD3 expression in HT29, HCT116, SW620, and DLD1 cells. Each lane was loaded with 25 μg protein, and β‐actin was used as a reference.

The protein expression level of PRKD3 was evaluated by Western blot analysis in four colorectal cancer cell lines: HT29, HCT116, SW620, and DLD1. As shown in Figure 5, *PRKD3* expression varied among the cell lines, with *DLD1* showing the highest level, followed by *SW620*, *HT29*, and *HCT116*.

Based on their relatively high *PRKD3* expression, *DLD1* and *SW620* were selected for subsequent functional experiments.

### 3.7 PRKD3 Knockdown Promotes Colorectal Cancer Cell Proliferation

**FIGURE 6:**
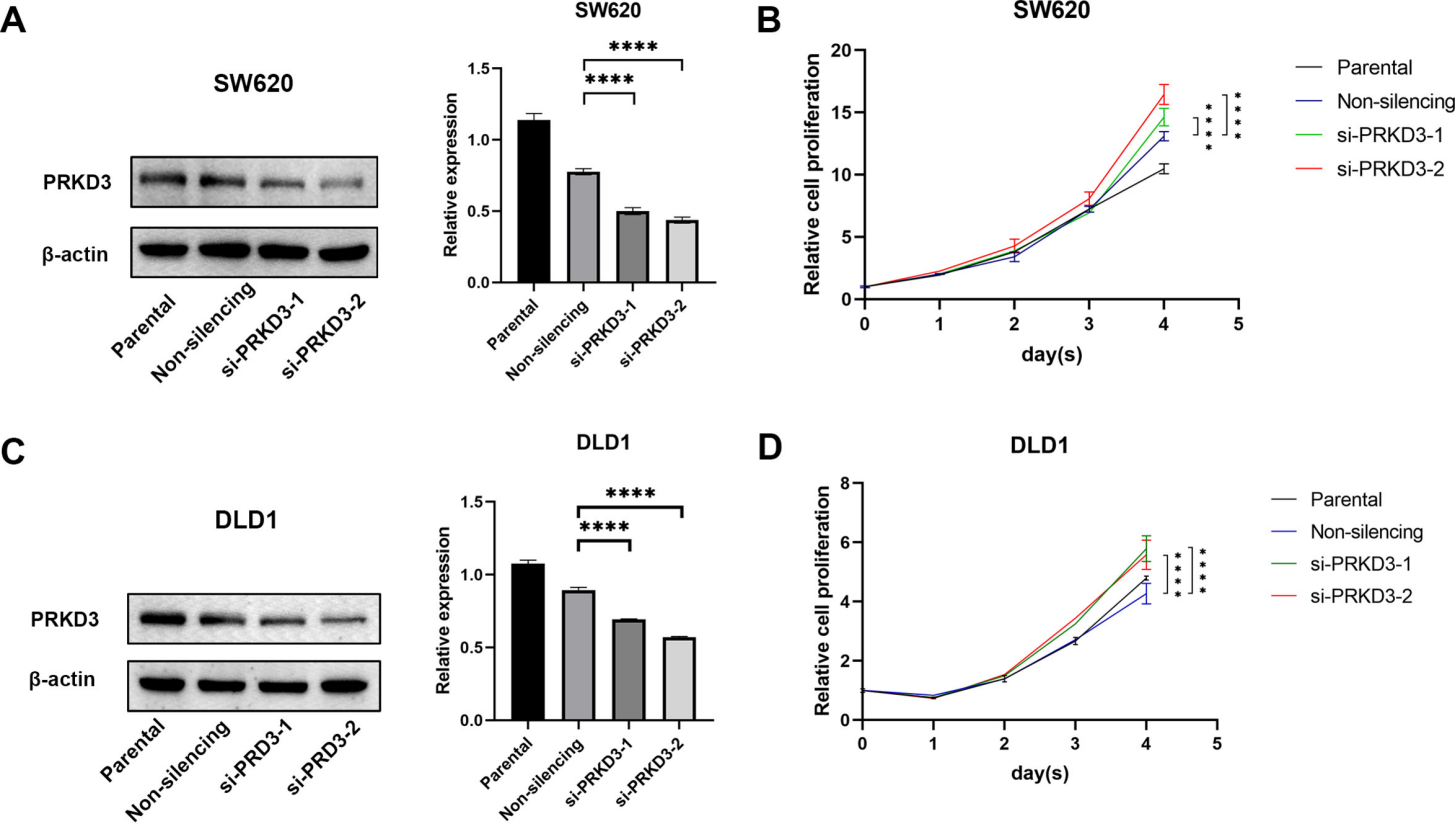
(A) PRKD3 expression changes 48 h post‐si‐RNA‐mediated knockdown in SW620 cells. (B) Assessment of cell proliferation post‐knockdown using a Cell Counting Kit‐8 assay, measuring OD450 values of SW620 cells at different time points. (C) PRKD3 expression changes 48 h post‐si‐RNA‐mediated knockdown in DLD1 cells. (D) Assessment of cell proliferation post‐knockdown using a Cell Counting Kit‐8 assay, measuring OD450 values of DLD1 cells at different time points. *p < 0.05; **p < 0.01; ***p < 0.001, ****p < 0.0001.

To investigate the functional role of PRKD3 in colorectal cancer, we performed siRNA‐mediated knockdown in DLD1 and SW620 cells using two independent siRNAs targeting PRKD3 (si‐PRKD3‐1 and si‐PRKD3‐2). Western blot analysis confirmed efficient knockdown of PRKD3 protein in both cell lines compared to the negative control siRNA (si‐NC) (Figure 6A,C).

Surprisingly, Cell Counting Kit‐8 (CCK‐8) assays demonstrated that knockdown of PRKD3 significantly promoted cell proliferation in both DLD1 and SW620 cells compared to the si‐NC group (*p* < 0.0001; Figure 6B,D).

## 4 Discussion

CRC is a major global health burden, with its incidence and mortality rates rising markedly in China. The pathogenesis of CRC is driven by the accumulation of genetic alterations. Following the multi‐hit model proposed by Fearon and Vogelstein, the classical adenoma‐carcinoma sequence has been well‐characterized. This sequence involves sequential mutations in key driver genes, including the tumor suppressors APC and TP53, and the oncogenes KRAS and PIK3CA. A pivotal early event is the loss of APC function, which leads to β‐catenin nuclear translocation and constitutive activation of the Wnt signaling pathway, thereby driving uncontrolled proliferation and tumor initiation.

Furthermore, comprehensive molecular profiling efforts, such as those by TCGA, have significantly refined our understanding of CRC by elucidating the roles of tumor mutational burden, microsatellite instability (MSI), and other regulatory mechanisms [8, 9]. At the pathological level, CRC typically originates from precancerous lesions, primarily conventional (tubular) adenomas and serrated polyps. The initiation of these lesions is driven by distinct molecular events: inactivation of the APC tumor suppressor gene in conventional adenomas and activating mutations in the BRAF oncogene in serrated polyps. Following these initial events, tumor progression proceeds along several well‐characterized molecular pathways, chiefly the chromosomal instability (CIN) pathway, the microsatellite instability (MSI) pathway, and the serrated neoplasia pathway [10]. Nevertheless, the current molecular frameworks do not fully elucidate the complexity of CRC pathogenesis. Consequently, identifying novel regulatory genes and dysregulated signaling pathways is imperative, as it may yield crucial mechanistic insights and unveil new therapeutic vulnerabilities for CRC.

As a serine/threonine kinase, PRKD3 is closely implicated in the occurrence and development of malignant tumors. For instance, Zhang et al. discovered that in gastric cancer, PRKD3 acts as an oncogene and stimulates gastric cancer development through the “p65/6‐phosphofructose‐2‐kinase/fructose‐2,6‐biphosphatase 3” pathway, suggesting its potential as a therapeutic target for gastric mucosal lesions [11]. In oral squamous cell carcinoma, Chen et al. found that PRKD3 correlates with metastasis and poor prognosis in patients, and it facilitates tumor progression by modulating KLF16, indicating its potential as a target for gene therapy in this cancer type [12]. In invasive breast cancer, Liu et al. demonstrated that PRKD3 is involved in various cancer‐related processes, such as cell cycle modulation and reduction of cell migration ability, through the analysis of phosphoproteome, interactome, and transcriptome [13]. Similarly, PRKD3 is overexpressed in prostate cancer, promoting the growth and survival of prostate cancer cells, and also facilitating the development of prostate cancer by up‐regulating adipose tissue production [14, 15]. In hepatocellular carcinoma, PRKD3 overexpression accelerates tumor progression and predicts a poor prognosis in patients following surgery [16]. Through enrichment analysis of signaling pathways, Yan et al. revealed that PRKD3 may facilitate hepatocellular carcinoma development and progression by activating mTORC1‐S6K1 and AKT/ERK1/2 signaling pathways, thereby promoting cellular proliferation and metabolic reprogramming [17].

Immunohistochemical (IHC) analysis demonstrated elevated PRKD3 protein expression in 68.7% of CRC tissues. In contrast, analysis of public transcriptomic data revealed lower PRKD3 mRNA levels in CRC compared to normal tissues, presenting an apparent discrepancy between protein and transcript abundance.

To investigate this inconsistency, the IHC experimental and scoring procedures were rigorously re‐examined, and the bioinformatic analysis was expanded using a larger dataset of matched samples. These validation steps confirmed the initial findings. Given this confirmed discrepancy, we considered several potential explanations based on a review of the relevant literature.

Firstly, data from the database may represent an average or general trend, as it is often pooled from a large number of samples. However, IHC experiments in our study may be influenced by factors such as sample selection and experimental procedures, leading to differences. Secondly, Jiang et al. conducted a study comparing the expression levels of protein molecules and corresponding mRNA in various tissues based on the Genotype‐Tissue Expression (GTEx) project. They found substantial differences between the transcriptome and proteome, indicating the existence of post‐transcriptional regulation [18]. Therefore, we hypothesize that PRKD3 may undergo post‐transcriptional regulation, contributing to the differential expression at the transcriptome and proteome levels. Self‐phosphorylation serves as a post‐transcriptional regulatory mechanism for PRKD3. Chen et al. investigated the role of PRKD3 in melanoma and revealed its ability to activate itself through self‐phosphorylation, inhibiting the reactivation of the mitogen‐activated protein kinase (MAPK) signaling pathway and enhancing the anti‐tumor effect of the RAF inhibitor RAF265 [19]. mRNA levels can be influenced by miRNA, RNA binding proteins, RNA degrading enzymes, etc., which may increase or decrease their expression levels [20]. In their investigation of neuroendocrine transdifferentiation in prostate cancer, Rayzel C et al. identified miRNA‐194 as a key post‐transcriptional regulator. By binding to the 3′ UTR of target mRNAs, miRNA‐194 induces mRNA degradation or translational repression, thereby relieving the suppression of genes such as IL‐8, Slug, and ZEB1. This process subsequently activates the ERK pathway and promotes neuroendocrine transdifferentiation in prostate cancer [21]. In a study on colorectal cancer, Ke Shen et al. identified a tumor‐suppressive miRNA, miR‐139‐5p, which competes for binding to the 3′‐UTR of target genes such as IGF1R, ROCK2, and RAP1B, thereby forming a competitive endogenous RNA (ceRNA) regulatory network. This mechanism enables bidirectional post‐transcriptional regulation, influencing not only protein expression but also tumor biological behavior [22]. Thus, we speculate that post‐transcriptional regulation plays a role in the inconsistency of PRKD3 expression. This also provides insights and directions for our future research. Subsequent studies will focus on testing this hypothesis through approaches such as proteasome inhibition, phosphoproteomics, or miRNA analysis. Lastly, we queried the CPTAC database for PRKD3 protein expression data in colorectal cancer tissues. Regrettably, to date, no detectable protein‐level expression data of PRKD3 in colorectal cancer tissues have been identified in this database.

Elevated PRKD3 protein expression was significantly associated with a favorable prognosis in patients with CRC. Although multivariate Cox regression analysis did not identify PRKD3 as an independent prognostic factor (*p* = 0.056), this borderline statistical significance suggests a potential, albeit non‐independent, prognostic role for PRKD3 in CRC. Collectively, these findings imply that PRKD3 may be involved in CRC pathogenesis and influence clinical outcomes. It is important to acknowledge, however, that the conclusions of this study are constrained by its relatively modest sample size and single‐center design, which may limit the generalizability of the results. Therefore, further validation of the prognostic significance of PRKD3 will require studies with larger sample sizes or multi‐center designs. Nevertheless, the findings may still offer some clinical relevance. Another possibility is the potential error in the histochemical staining process. Additionally, subjective scoring of staining can introduce human error.

An exploratory analysis revealed a borderline significant correlation between PRKD3 protein expression and MMR status (pMMR vs. dMMR; *p* = 0.087). The four dominant MMR proteins are MLH1, MSH2, MSH6, and PMS2. Mismatch repair deficiency (dMMR) is defined as loss of ≥ 1 major MMR protein, while proficient MMR (pMMR) indicates intact expression of all four proteins. MMR status is clinically used for hereditary CRC detection, broadly categorized as follows: (1) polyposis‐associated syndromes (e.g., FAP, MAP, hamartomatous polyposis), and (2) non‐polyposis syndromes (e.g., Lynch syndrome) [23]. Database analysis showed a significant association between PRKD3 expression levels and polyp history in CRC (*p* < 0.05). These exploratory results suggest a potential relationship between MMR status and polyp history in CRC patients, though larger validation cohorts are required to substantiate this observation.

In this study, we established a PRKD3 knockdown model using siRNA transfection in colorectal cancer cell lines, and investigated its impact on cell proliferation. Our findings revealed that cells with PRKD3 knockdown exhibited significantly higher proliferation abilities compared to the control group. These results suggest a potential inhibitory role of PRKD3 in the carcinogenic processes and development of colorectal cancer.

Currently, there is limited research on PRKD3 expression in CRC, and its precise role and associated molecular mechanisms in this cancer type require further investigation.

## 5 Conclusion

This study identified abnormal PRKD3 expression in CRC compared to normal tissues. Notably, a potential association was observed between elevated PRKD3 expression and improved patient prognosis. This preliminary finding provides a basis for further investigation into its role as a candidate biomarker. Cellular experiments further demonstrated that elevated PRKD3 expression can inhibit CRC cell proliferation. Collectively, these findings suggest the potential utility of PRKD3 as a molecular marker for CRC, with possible applications in disease typing, clinical staging, and prognosis assessment. This molecule could be explored as a potential target for future therapeutic strategies.

Furthermore, this research represents a preliminary experimental exploration of PRKD3 expression in CRC, primarily utilizing clinical samples. Future studies could consider integrating molecular and clinical indicators to construct predictive models, while incorporating animal models to investigate underlying mechanisms, and further exploring the PRKD3 regulatory network through approaches such as transcriptomics or phosphoproteomics.

## Author Contributions

**Bin Zhang:** data curation, formal analysis, writing – original draft, validation, software, investigation. **Chen Chang:** investigation, writing – original draft, validation, software, formal analysis, data curation. **Jingli Chen:** data curation, software. **Yue Cao:** data curation, software. **Yu Chang:** data curation. **Lele Liu:** data curation. **Wensheng Li:** conceptualization, methodology, writing – original draft, supervision. **Guorong Wang:** conceptualization, funding acquisition, methodology, project administration, resources, writing – review and editing.

## Funding

This work was supported by the Innovation ability support plan of Science and Technology Department of Shaanxi Province–Science and Technology Innovation Team (2020TD‐048), the Scientific and Technological Personnel Support Program of Shaanxi Provincial People's Hospital (2021LJ‐08), the Natural Science Foundation of Shaanxi Province (2023‐JC‐QN‐0851).

## Ethics Statement

This study was conducted in accordance with the Declaration of Helsinki, and approved by the Ethics Committee of Shaanxi Provincial People's Hospital (No. 2023‐R057). Informed consent was not required for this retrospective study.

## Consent

Patient consent was waived as written informed consent had been obtained from the patients when undergoing surgeries in the hospital.

## Conflicts of Interest

The authors declare no conflicts of interest.

## Data Availability

The data that support the findings of this study are available from the corresponding author upon reasonable request.
